# A Lightweight Exoskeleton-Based Portable Gait Data Collection System †

**DOI:** 10.3390/s21030781

**Published:** 2021-01-24

**Authors:** Md Rejwanul Haque, Masudul H. Imtiaz, Samuel T. Kwak, Edward Sazonov, Young-Hui Chang, Xiangrong Shen

**Affiliations:** 1 Department of Mechanical Engineering, The University of Alabama, Tuscaloosa, AL 35487, USA; mhaque2@crimson.ua.edu; 2 Department of Electrical and Computer Engineering, Clarkson University, Potsdam, NY 13699, USA; mimtiaz@clarkson.edu; 3 School of Biological Sciences, Georgia Institute of Technology, Atlanta, GA 30318, USA; skwak@gatech.edu (S.T.K.); yh.chang@ap.gatech.edu (Y.-H.C.); 4 Department of Electrical and Computer Engineering, The University of Alabama, Tuscaloosa, AL 35487, USA; esazonov@eng.ua.edu

**Keywords:** exoskeleton, wearable sensors, gait measurement

## Abstract

For the controller of wearable lower-limb assistive devices, quantitative understanding of human locomotion serves as the basis for human motion intent recognition and joint-level motion control. Traditionally, the required gait data are obtained in gait research laboratories, utilizing marker-based optical motion capture systems. Despite the high accuracy of measurement, marker-based systems are largely limited to laboratory environments, making it nearly impossible to collect the desired gait data in real-world daily-living scenarios. To address this problem, the authors propose a novel exoskeleton-based gait data collection system, which provides the capability of conducting independent measurement of lower limb movement without the need for stationary instrumentation. The basis of the system is a lightweight exoskeleton with articulated knee and ankle joints. To minimize the interference to a wearer’s natural lower-limb movement, a unique two-degrees-of-freedom joint design is incorporated, integrating a primary degree of freedom for joint motion measurement with a passive degree of freedom to allow natural joint movement and improve the comfort of use. In addition to the joint-embedded goniometers, the exoskeleton also features multiple positions for the mounting of inertia measurement units (IMUs) as well as foot-plate-embedded force sensing resistors to measure the foot plantar pressure. All sensor signals are routed to a microcontroller for data logging and storage. To validate the exoskeleton-provided joint angle measurement, a comparison study on three healthy participants was conducted, which involves locomotion experiments in various modes, including overground walking, treadmill walking, and sit-to-stand and stand-to-sit transitions. Joint angle trajectories measured with an eight-camera motion capture system served as the benchmark for comparison. Experimental results indicate that the exoskeleton-measured joint angle trajectories closely match those obtained through the optical motion capture system in all modes of locomotion (correlation coefficients of 0.97 and 0.96 for knee and ankle measurements, respectively), clearly demonstrating the accuracy and reliability of the proposed gait measurement system.

## 1. Introduction

With the rapid technological advances in electronics, software, and robotic actuation, the development of wearable devices for the assistance of human locomotion has been a very active area of robotics research in recent years. Such devices are able to assist human users’ locomotion through joint-level assistance (exoskeletons and orthoses, such as), or restore the lost lower-limb functions by functioning as artificial limbs (robotic prostheses, such as). The incorporation of a power source (usually a battery) and computing and electronic components enables these wearable assistive devices to operate in a fully independent (i.e., untethered) way, providing the potential of extensive real-world use in people’s daily life.

Despite the readiness of the robot hardware, the effectiveness of wearable robot-provided motion assistance is severely limited by the performance of the robot control system, especially when complex daily-living scenarios are involved. Effective identification of human motion intent often involves the implementation of a pattern recognition algorithm to classify the current and desired motion modes. Effective control of joint motion or joint assistance requires well-regulated joint power delivery in response to the user’s biological limb movements. Both of these critical functions are established on the quantitative understanding of human locomotion, for which the collection of related gait data is indispensable. However, existing approaches for gait data collection suffer from a number of weaknesses that affect their performances (especially in complex daily-living scenarios), which constitutes a major challenge in assistive robot controller development.

### 1.1. Prior Works on Gait Data Collection

Marker-based optical motion capture [1,2] is the most extensively used approach of gait measurement, with very high precision [3] (<1 mm in position measurement [4]). As such, marker-based optical motion capture is generally accepted as the gold standard in human motion measurement. When a sufficient number of markers are attached to the torso or a limb segment, a marker-based system is able to accurately locate the torso or limb segment (both position and orientation) in the three-dimensional space, providing a wealth of information on the 3D human movement. Despite the performance advantages, the marker-based systems’ weaknesses are also obvious. Multiple cameras need to be securely mounted in the workspace, making the system expensive to acquire and time-consuming to install. A significant amount of time is required to apply the markers to each individual test participant, making the experiments difficult to set up. The requirement of applying markers to bony landmarks (instead of on clothes) further exacerbates the problem. Substantial efforts are also needed after the experiments to extract the useful information (e.g., joint angles) from the raw data. Last but not least, marker-based motion capture is largely confined to the typical gait laboratory setting, making it nearly impossible to collect gait data in real-world daily-living scenarios (e.g., outdoor environments).

To address the problems with the marker-based motion capture, there were two major technical approaches introduced in recent years, including markerless optical motion capture [5,6,7,8], and inertial motion capture. A markerless motion capture system does not require the application of reflective markers, making the experimental setup much easier. Markerless systems are also less expensive. However, the accuracy of markerless systems is in general significantly lower than marker-based systems [9,10], and the measurement is also sensitive to environmental factors.

Inertia motion capture is fundamentally different from the optical motion capture techniques described above, as it uses acceleration and angular velocity measurements to obtain the torso and limb orientations. Advanced processing techniques, such as Kaman Filters, have been explored to reduce measurement error (within 2° root mean square as reported in [11]). Inertia sensors, also known as inertia measurement units (IMUs), can be attached to different parts of the human body and facilitate the gait measurement outside gait laboratories (when portable wireless IMUs are used). As the major weakness of inertia motion capture, the measurement error tends to accumulate due to the use of integration techniques. Further, it is difficult to securely attach the inertia measurement sensors to the human torso and limbs with proper alignment, which affects the reliability of the measurement and causes additional measurement errors due to the sensors’ movement relative to the human body.

For the gait data collection, the variables of the strongest interest to the researchers are usually the angles of the lower-limb joints, primarily the knee and ankle. Trajectories of these joint angles can be used as the inputs to intent recognition algorithms [12,13]. Joint angle trajectories measured on healthy individuals may also serve as the setpoints for the control of wearable robots such as robotic prostheses. The aforementioned methods of motion capture, in general, obtain the joint angle by comparing the spatial orientations of the corresponding limb segments, which involves complex and time-consuming data processing. In comparison, direct measurement of joint angle can be conducted with wearable goniometers. By aligning the rotation axis of the goniometer with the corresponding biological joint, the sensor output directly reflects the joint displacement (i.e., no complex data processing needed). The most commonly used type of goniometer is the traditional rotary potentiometer, and magnetic encoders and fiber-optical sensors can also serve as the goniometers for joint measurement purposes [14,15,16]. Note that, when portable goniometers are used, these devices can be used for mobile gait measurement without being limited to the lab environments, facilitating the gait data collection in people’s real-world daily-living scenarios. Despite the advantages, joint goniometers, similar to the aforementioned inertia sensors, are wearable sensors in nature, requiring secure attachment to the human body with proper alignments. Such requirement is difficult to meet with the traditional strap-based mounting method, which tends to shift over the surface of human limbs during movement.

### 1.2. Lightweight Exoskeleton-Based Gait Data Collection

Motivated by the limitations imposed by the existing gait measurement systems, the authors developed a novel lightweight exoskeleton-based system to collect gait data without being limited to the traditional gait lab environment. Compared with the aforementioned wearable sensors, the proposed exoskeleton-based approach provides a new method to integrate sensors of multiple modalities (goniometers, inertia sensors, and pressure sensors) into a portable data collection system. The advantage of this integrative approach is two-fold. From a mechanical perspective, the use of a single lightweight exoskeleton eliminates the need of mounting multiple individual sensors to the human body and allows the sensors to be securely and stably attached to the human limb segments with consistent alignment. From an electronic and data-processing perspective, merging the multiple sensor inputs into a single microprocessor-based data collection system enables all sensors to be wire-connected, providing consistent and high-quality data transfer unaffected by the electromagnetic interference common in outdoor environments. Further, the data from multiple sources can be easily synchronized to facilitate the subsequent analysis. 

Using an exoskeleton for gait measurement also comes with significant challenges. The device must be lightweight to avoid excessive burden to the user. The device must also have sufficient adjustability to fit different users with a reasonable range of adjustment on height and limb contour. More importantly, the device’s interference to the user’s biological joint motion must be minimized to avoid affecting his/her natural full-body movement. To meet these requirements, the gait measurement exoskeleton in this paper features a lightweight aluminum frame structure enhanced with a simple yet effective height adjustment mechanism and a unique 2-degree-of-freedom instrumented joint design. Inertial measurement units (IMUs) can be securely attached to the exoskeleton at multiple locations to enable the measurement of the thigh and shank’s spatial orientations. A force-sensing resistor (FSR)-based plantar pressure measurement membrane is integrated with the exoskeleton’s footplate, providing a reliable measurement of important gait events such as heel strike and toe-off, as well as the shift of the pressure center during gait. All sensor signals are routed to a microcontroller for data logging and storage. The paper is organized as follows: Section 2 presents the details of the mechanical design of the exoskeleton and the development of the data collection system. Section 3 presents the results obtained in the human experiments that demonstrate the validity and performance of this unique gait data collection system. Section 4 presents a discussion of the research in this paper and the future works, and Section 5 presents the conclusions of this work.

## 2. Methods

### 2.1. Exoskeleton Design

**Figure 1:**
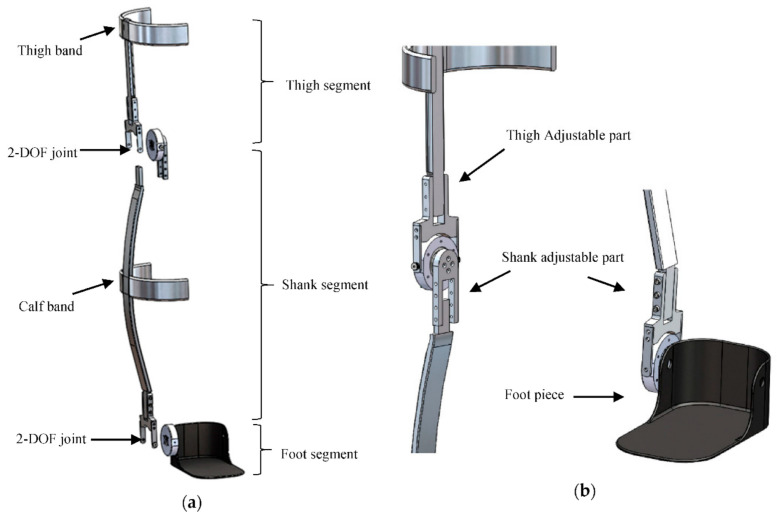
(**a**) Exploded view of 2-Degree-of-Freedom (DOF) joints of the Measurement exoskeleton system (developed for right leg [17]; (**b**) Detailed view height adjustment mechanism [17].

The key objectives of the Exoskeleton design [17] include reducing the device weight, generating a compact profile, and providing a comfortable user interface to minimize the interference with the user movement while supporting a sensor array for accurate lower-limb motion capture. The mechanical structure of the instrumented exoskeleton consists of three segments, including a thigh segment, a shank segment, and a foot segment. These segments are connected by two instrumented joints to measure the movement of the corresponding biological joints as shown in Figure 1a. In order to reflect the range of motion of the knee and ankle about the sagittal and frontal plane, an additional degree of freedom was incorporated into the joint design. This additional degree of freedom allows the unrestricted movements in the frontal plane without interfering with the measurement of the joint sensor. 

Traditional joints in the lower-limb exoskeletons are mostly 1-DOF, which imposes significant constraints on the user’s leg movement. The constraint is especially severe for the ankle, which is a 3-DOF joint in nature [18,19]. Among the rotations with respect to the three axes, flexion–extension is the major mode of motion, and its angular displacement is usually an important variable to be measured in walking experiments. However, inversion–eversion of the ankle (associated with the lateral tilting of the foot) is also obvious in human walking. Disabling such ankle movement would significantly alter a human’s natural walking behavior, especially during turning movements [20]. For the knee, although its movement in the sagittal plane is the dominant mode of motion, a 1-DOF joint in the exoskeleton would still affect the user’s comfort in walking, especially considering the change of the thigh volume and shape in different phases of a walking gait cycle.

To minimize the constraint imposed by the exoskeleton on the user’s nature joint movement, the authors developed a unique 2-DOF joint that combines a major measurement DOF (instrumented with an absolute magnetic encoder) and a minor passive DOF (to allow natural joint movement and improve user comfort). The structure of the 2-DOF joint is shown in Figure 1a.

To establish a linkage between two joints, a thin (5-mm thickness) aluminum bar was used. The shape of the linkage can be adjusted using standard orthotic bending irons to fit and align with the user’s calf curvature. The connectors between the aluminum bar and either joint provide a measure of height adjustability as shown in Figure 1b, with a range of approximately 7.6 cm. This simple yet effective adjustment mechanism enables the device to fit subjects at different heights in a configuration that ensures the joint sensor is fixed on-axis with the rotation of the measured joint.

For the joint sensor to reliably measure the corresponding joint angle, it must remain aligned with the axis of rotation. Given the curvature of the thigh and calf, however, the device may misalign after repetitive motion. To address this issue, two aluminum bands for the thigh and the calf were incorporated along with webbing straps coupled with tri-glide slides and buckles, ensuring minimal sensor shift relative to the user’s body. The shape of the thigh/calf band can also be adjusted to fit users with different limb form factors using orthotic bending irons.

The ankle joint was attached to a carbon fiber foot segment embedded underneath the user’s shoe sole, providing a fixed frame of reference to measure the angular position of the ankle joint.

### 2.2. Sensors, Interfacing, and Data Acquisition

The exoskeleton system consists of a set of sensors, including two rotary magnetic encoders, two force-sensing resistors (FSR), and two 9-DOF Inertial Measurement Units (IMU) along with data acquisition electronics powered by a 3.7 V Li-polymer battery of 300 mAh capacity. This system also incorporated STM32L476RG, a Cortex-M4 Ultra-low-power ARM processor (ST Microelectronics, Geneva, Switzerland) with an 80 MHz CPU at 39 µA/MHz; a 16 GB micro-SD card to store data sampled at 1 kHz by the MCU (microcontroller); and a micro-USB interface to control data collection, access sensor signals stored in the SD card, update MCU timestamp, recharge the battery, and upload the firmware.

**Figure 2:**
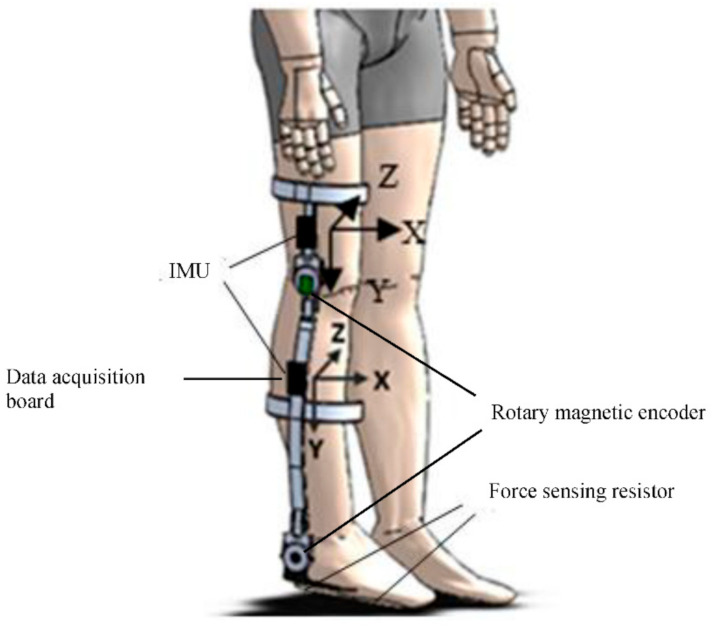
The placement of sensors and data acquisition board [17].

The rotary magnetic encoders AS5145 (AMS AG, Unterpremstätten, Austria) were placed in the 2-DOF joints to measure the angular position of the knee and ankle. After installation, the joint position readings were calibrated to ensure proper zero positions for the joints. Note that no calibration is needed for other sensors after installation. Two FSRs (FS406, Interlink Electronics, Camarillo, CA, USA) were embedded under the shoe sole to measure the heel and ball pressures under the foot. Protective plastic sheaths were placed around two FSRs with a layer of insulating tape and embedded into a shoe insole. Each FSR has a 39.6-mm square active area, which allows it to provide effective measurement for a wide range of participants with different foot sizes. One IMU was embedded into the circuit board, which was mounted on the exoskeleton (shank segment) below the knee joint, and the other was mounted on the thigh segment. All IMUs were positioned at a configuration to have the *y*-axis perpendicular to the ground, the *x*-axis parallel to the ground toward body movements and the *z*-axis away from the body. The placement of all the sensors and the data accusation board is illustrated in Figure 2.

**Figure 3:**
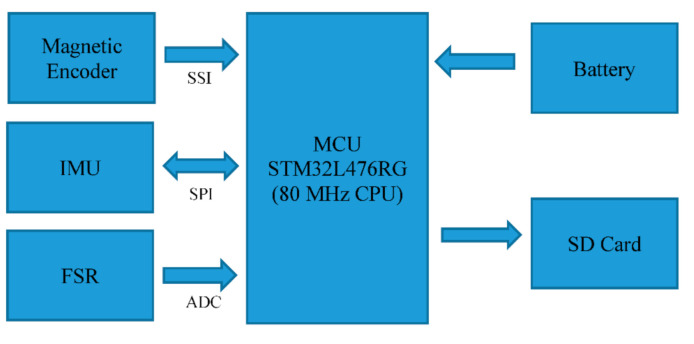
Sensor interfacing and data acquisition electronics.

Two magnetic encoders were interfaced with the Microcontroller Unit (MCU) through two Serial Synchronous Interfaces (SSI), a standard serial interface between an absolute position sensor and a controller. The encoders can measure acute angles over a full turn of 360 degrees with 14 bits of resolution. The polymer thick film FSR is capable of measuring pressure utilizing its property of decreasing resistance with the increase in the applied force on its active surface. A resistive divider was formed by each FSR and a 500 Ω resistor and applied to op-amp-based voltage followers. The Op-amp output of FSRs was interfaced with two ADC channels of the MCU (with 12 bits of resolution). The motion tracking was performed by the IMUs (MPU-9250, InvenSense Inc., San Jose, CA, USA), each combining a 3-axis gyroscope and a 3-axis accelerometer. The accelerometer and gyroscope of the modules were configured to have a ±8 g and ±2000 dps measurement range, respectively, with 16 bits of resolution. The IMUs were interfaced with the MCU through two SPI interfaces. A block diagram of the sensors’ interfacings is shown in Figure 3.

### 2.3. Experimental Procedure

Three healthy volunteers (details summarized in Table 1) with no physical and cognitive abnormalities, participated in this test. All volunteers gave informed written consent before participation according to a protocol. The protocol of the experiment was approved by the Georgia Institute of Technology Institutional Review Board.

**Figure 4:**
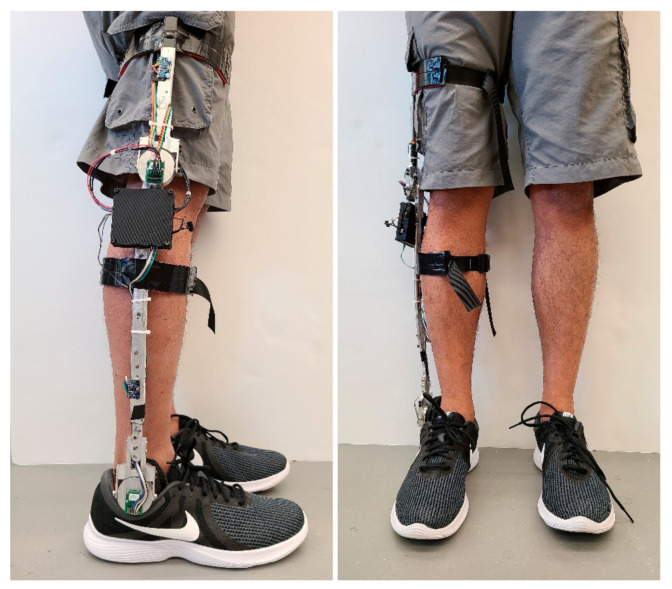
Prototype of the measurement exo-skeleton.

In a laboratory setting of an eight-camera Vicon motion capture system (120 Hz, Vicon Motion Systems, Oxford, UK), the collection of samples from healthy individuals was performed and the performance and suitability of the system were evaluated. First, each subject was asked to wear the exoskeleton device, shown in Figure 4. Reflective markers were placed on the subject’s second metatarsophalangeal joint, lateral malleolus, lateral condyle, anterior superior iliac spine, posterior superior iliac spine, shank segment, and thigh segment. Before starting the data collection, the subjects were asked to walk normally for 5–10 min to get comfortable with the device. After that, they performed the following locomotives activities sequentially: (a) repeated sit-to-stand/stand-to-sit for 5 times, (b) Level ground walking in self-selected moderate and fast cadence, (c) Treadmill walking in four incremental speeds: starting at 0.5 m/s to 1.25 m/s with an increment of 0.25 m/s, (d) Treadmill walking in gradual speed increase from 0.5 m/s to 1.25 m/s. These activity modes were chosen as they are common in people’s daily life, relatively easy to implement and measure in the lab, and involve significantly different limb/joint movements (small-range cyclical movements during walking, and big-range, transitional movements in sit-to-stand/stand-to-sit motion). All these individual activities had a maximum duration of 1 min. All participants repeated the same activity sequence after taking a rest for 10–15 min. The entire laboratory session was videotaped by an iON contour video camera at a 60 fps capture rate. In a smartphone application (aTimeLogger—Time Tracker), the start–end timestamp of each activity was marked. The Vicon system, video camera, and the smartphone were time-synchronized with the exoskeleton by sending the same internet timestamp to all three devices. After the experiment session, volunteers were asked to provide feedback on the acceptability of the exoskeleton system in terms of comfortability, longer-term usage, and effect on mobility.

### 2.4. Data Collection and Processing 

Both the exoskeleton system and the eight-camera motion analysis system recorded the lower limb kinematics data simultaneously. The motion capture data were filtered a zero-phase lag fourth-order Butterworth low-pass filter with a 10 Hz cutoff frequency. The recorded exoskeleton sensor signals were first processed by a dedicated MATLAB script for noise removal. A second-order low-pass Butterworth filter with an empirically selected cutoff frequency of 10 Hz was then applied to individual sensor signals. 

## 3. Results

The experimental results are presented in the following subsections.

### 3.1. Ankle Joint Measurement Evaluation

**Figure 5:**
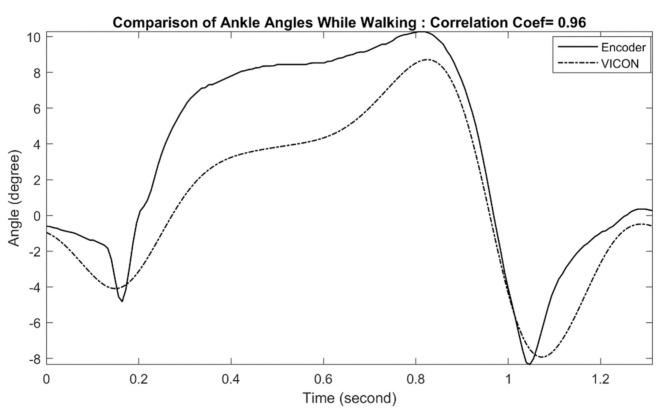
Comparison of Exo-skeleton’s ankle angle measurement with reference motion capture during walking. In this data, the correlation coefficient was calculated at 0.96.

An example of measured ankle joint angles during treadmill walking is shown in Figure 5. The figure shows a little deviation in exoskeleton ankle measurement in comparison with the reference optical motion capture measurement, with a correlation coefficient of 0.96. This result indicates that the exoskeleton can measure ankle position with a negligible amount of error. All other walking trials (level walking and treadmill walking) with other subjects (male and female with different heights) and at different speeds show similar performance. The calculated correlation coefficients for all waking trials are summarized in Table 2.

**Figure 6:**
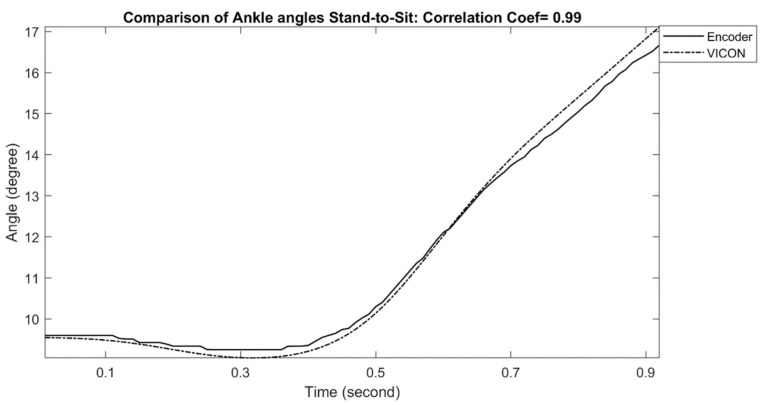
Comparison of Exo-skeleton’s ankle angle measurement with reference motion capture during stand to sit. In this data, the correlation coefficient was calculated at 0.99.

**Figure 7:**
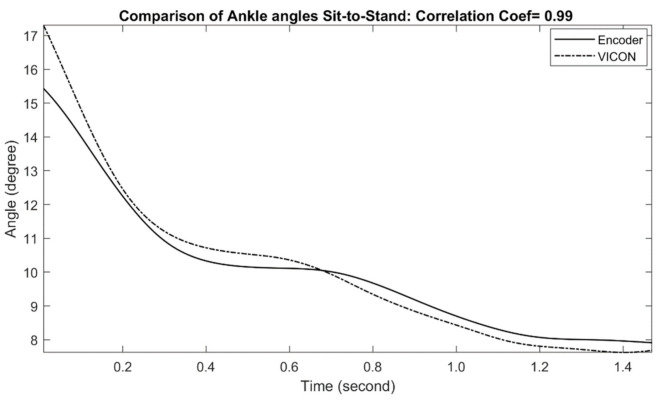
Comparison of Exo-skeleton’s ankle angle measurement with reference motion capture during sit-to-stand. In this data, the correlation coefficient was calculated at 0.99.

The comparisons of measured ankle joint angles with a reference motion capture system during stand-to-sit and sit-to-stand activities are shown in Figure 6 and Figure 7, respectively. For stand-to-sit activity, the exoskeleton ankle measurement shows a very good match in comparison with the reference optical motion capture measurement, with a correlation coefficient of 0.99. Similarly, sit-to-stand/stand-to-sit activities also show good results in comparison, with a correlation coefficient of 0.99, indicating excellent performance. The calculated correlation coefficients for all stand-to-sit and sit-to-stand trials with other participants are summarized in Table 2.

### 3.2. Knee Joint Measurement Evaluation

**Figure 8:**
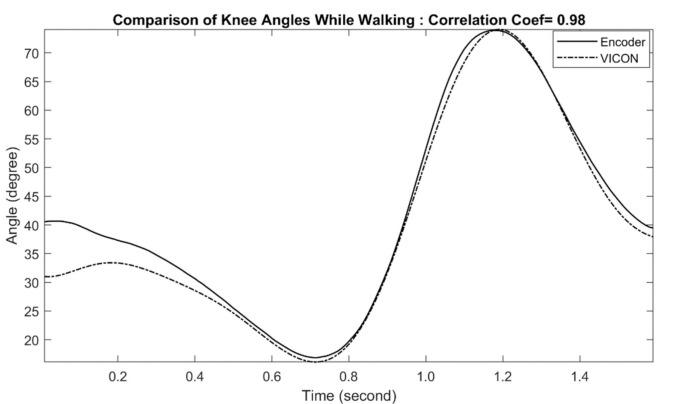
Comparison of Exo-skeleton’s knee angle measurement with reference motion capture during walking. In this data, the correlation coefficient was calculated at 0.98.

The comparison of exoskeleton knee joint measurement with motion capture measurement during the treadmill walking is shown in Figure 8. The comparison shows very little deviation between the two measurement approaches, particularly during the early stance of the gait cycle (correlation coefficient of 0.98). The performance of the subsequent trials at different speeds is summarized in Table 3. In all trials, both exoskeleton and motion capture approaches yield similar results.

**Figure 9:**
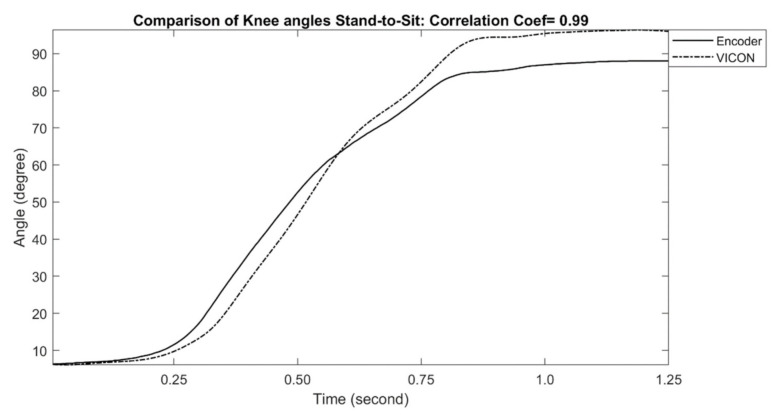
Comparison of Exo-skeleton’s knee angle measurement with reference motion capture during stand-to-sit. In this data, the correlation coefficient was calculated at 0.99.

**Figure 10:**
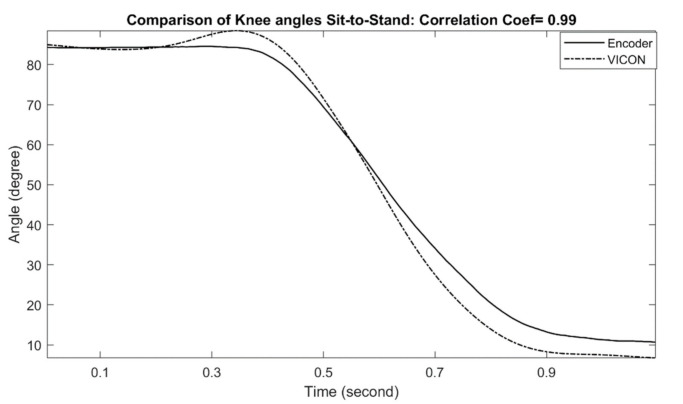
Comparison of Exo-skeleton’s knee angle measurement with reference motion capture during sit-to-stand. In this data, the correlation coefficient was calculated at 0.99.

The examples of measured knee joint angles during stand-to-sit and sit-to-stand activities are shown in Figure 9 and Figure 10, respectively. For stand-to-sit activity, the exoskeleton knee measurement shows an excellent match in comparison with reference optical motion capture system measurement (correlation coefficient of 0.99). Likewise, sit-to-stand activity shows a similar match in comparison, and the correlation coefficient was determined as 0.99, which can be interpreted as an excellent performance. The calculated correlation coefficients for all stand-to-sit and sit-to-stand trials with other participants are summarized in Table 3.

### 3.3. Plantar Pressure Measurement and Gait Events Detection

**Figure 11:**
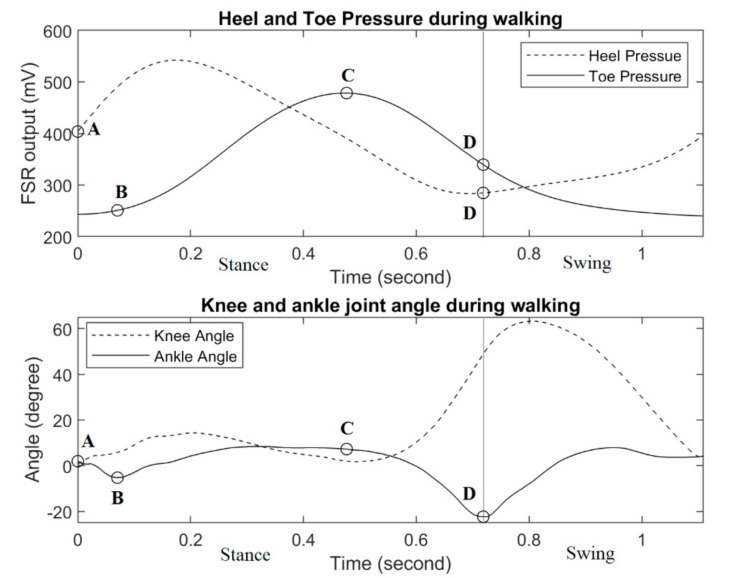
Responses of force-sensing resistors at important gait events, along with the corresponding ankle and knee joint trajectories. Point (A): Initial ground contact event, (B) Forefoot contact event, (C) Push-off event and (D) Toe-off event.

To identify the gait events, both the heel and the toe sensor’s responses are shown in Figure 11, along with the corresponding ankle and knee joint trajectories. Typically, a gait cycle can be divided into two phases: stance and swing. The stance is the phase during which the foot is in contact with the ground and it covers approximately 60% of the gait cycle, while the remaining 40% is the swing phase. At the beginning of the stance, the heel contacts the ground surface for the first time (marked as point A), which is known as the initial contact event. Subsequently, heel pressure starts to increase while the toe pressure stays minimal (which can be seen in Figure 11). The forefoot contact event starts (point B) when the metatarsal heads contact the ground for the first time, and the toe pressure starts to increase. Then, the push-off event (point C) begins when the metatarsal heads and the toes are the only support in contact with the ground, and as a result, the toe pressure sensor shows maximum reading. Finally, the toe-off event (point D) is considered the transition between the stance and the swing. During the swing phase, both heel and toe pressures stay minimal.

Heel strike and toe-off are the two most crucial events in identifying the transition between the stance and swing phases. The heel strike determines the start of the stance phase and therefore the end of the swing phase. On the other hand, toe-off determines the end of the stance phase and the beginning of the swing phase.

### 3.4. Three Dimensional Kinematic Information

**Figure 12:**
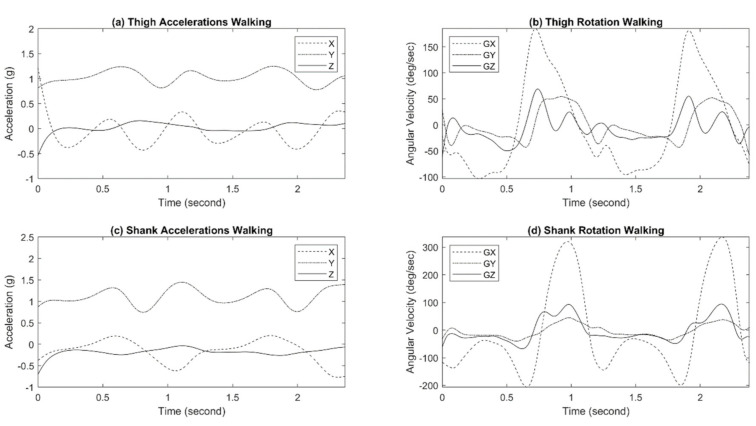
Exo-skeleton inertia measurement unit (IMU) responses during walking.

**Figure 13:**
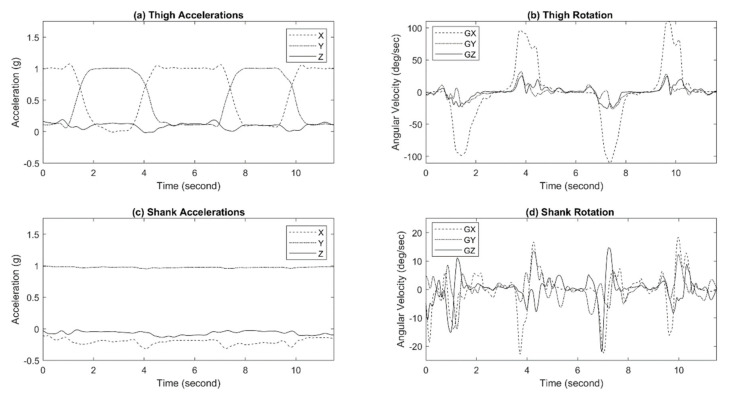
Exo-skeleton IMU responses during sit-to-stand/stand-to-sit motion.

The responses of the shank IMU and the thigh IMU during walking and sit-to-stand/stand-to-sit motion are shown in Figure 12 and Figure 13, respectively. All IMUs were positioned at a configuration to have the *y*-axis perpendicular to the ground, the *x*-axis parallel to the ground toward body movements, and the *z*-axis away from the body.

## 4. Discussions

In this paper, an instrumented wearable exoskeleton for gait measurement of human locomotion was developed. It is a portable, low-cost, durable, and lightweight device to collect lower-limb biomechanical information of the wearer with the capability of measurement in indoor and potentially in outdoor environments. The 2-DOF joints of the device enabled unrestricted knee and ankle movements in the frontal plane, which significantly improved the comfort in wearing. This is further verified by the test subjects, as they were comfortable wearing the device on their lower limbs and responded positively to the possibility of extended use. The adjusting mechanism of the device provided height adjustability with a range of approximately 7.6 cm in the current device, which can be further increased by customizing the link between the joints. In our experiments, a single device setup was used for all three subjects of different heights (170–182 cm). The simple yet effective adjustment mechanism enables the device to fit subjects at different heights in a configuration that ensures the joint sensor is fixed on-axis with the rotation of the measured joint, which opens the door to a promising universal plug-and-play gait analysis opportunity.

The evaluation of the exoskeleton system in a reference motion capture environment demonstrates acceptable accuracy in measuring lower limb joint angles for multiple activities performed by the participants. For all trials in various conditions, the comparison shows slight or no deviation between the two measurement approaches. The comparison shows slight or no deviation between the two measurement approaches. The average ankle joint angle correlation coefficients during ground walking, treadmill walking, stand-to-sit, and sit-to-stand were 0.97, 0.96, 0.98, and 0.98, respectively. Similarly, the average knee joint angle correlation coefficients during ground walking, treadmill walking, stand-to-sit and sit-to-stand were 0.96, 0.96, 0.98 and 0.98, respectively. The lowest correlation coefficient was recorded as 0.95 in ankle measurement in just one trial, which can be regarded as acceptable since the average values of the correlation coefficient showed even higher accuracy even if walking speeds or subjects were different. Therefore, it is expected that the exoskeleton can measure lower limb joint angles during gait with good accuracy independent of activities, walking speeds and subjects.

The shoe-embedded heel and toe force-sensing resistors of the exoskeleton may potentially be used for gait studies as such gait events detection, pending further validation. Four main gait events, such as initial heel contact, forefoot contact, push-off, and toe-off are identifiable from the response patterns of the heel and toe sensors. In this study, the gait event detection was solely based on signal patterns analysis of the heel and toe sensor rather than the quantification of plantar pressure. Future studies may include the validation of the heel and toe pressure sensor with the reference force plate by comparing the ground reaction force.

Additionally, the shank and thigh IMUs of the exoskeleton system provide an estimate of three-dimensional accelerations and angular velocities in the lower limb gait analysis.

The successful testing of the exoskeleton system on three volunteers in a motion capture environment suggests possible implementation in prostheses responding to the intention of the wearer. However, this is limited by the sampling of only three subjects. To further pursue this, performance evaluation with varying body sizes and gait patterns is necessary, requiring both a larger sample size and testing on disabled and elderly subjects. The foreseen potential use of this exoskeleton system is primarily the development of a personalized adaptive controller with automatic parameter tuning. Additionally, the exoskeleton may be used in conjunction with other types of sensors (such as EMG sensors) to obtain more comprehensive gait data sets. Two exoskeletons, worn on both left and right legs, may provide simultaneous bilateral gait measurement to quantify gait symmetry. Synchronization strategies, such as those developed by Coviello et al. [21], can be used to synchronize the multiple sensing devices and better relate all the measurements. As such, the use of the exoskeleton system may potentially expand beyond the prosthesis control application and provide gait measurement for individuals suffering from other mobility-related pathologies such as Parkinson’s Disease.

## 5. Conclusions

This paper presents a novel exoskeleton-based portable gait data collection system. Unlike the existing wearable sensors for gait measurement purposes, the use of an exoskeleton eliminates the need for attaching individual sensors separately, which simplifies the sensor attachment and allows the sensors to be attached securely and stably with consistent alignment. As the basis of the data collection system, a lightweight exoskeleton was developed, which incorporates a 2-DOF joint design to minimize the interference to the users’ natural joint movement and a simple yet effective adjustment mechanism to fit users at different heights. Mounted on the exoskeleton frame are multiple sensors of different types, including joint-angle magnetic sensors, IMUs, and plantar pressure FSRs. The performance of the proposed gait data collection system was experimentally tested on three healthy subjects, with an eight-camera optical motion capture system providing benchmark measurements of the joint angles for the validation purpose. The measurement results provided by the exoskeleton closely matched those obtained through optical motion capture, with high correlation coefficients for both the knee (0.97) and the ankle (0.96). The quantitative results from the experiments, combined with the test subjects’ feedback on the comfort of wearing, clearly demonstrated that the proposed exoskeleton system is able to provide high-accuracy gait measurement with adequate user comfort for extended use.

## Figures and Tables

**Table 1:** The Particulars of the volunteers.

Volunteer	Gender	Age (Years)	Height (cm)	Past Injuries
1	Male	28.1	174	None
2	Female	26	170	None
3	Male	21.1	182	None

**Table 2:** The summary of Ankle angle measurement comparison (exoskeleton vs. Vicon).

Activity	Speed (m/s)	Correlation Coefficient		
		Subject 1	Subject 2	Subject 3
Level ground Walking	Self-selected	0.97	0.96	0.97
Treadmill walking	0.5	0.97	0.96	0.97
	0.75	0.96	0.96	0.96
	1	0.95	0.96	0.96
	1.25	0.96	0.97	0.96
	Gradual increase (0.5–1.25)	0.97	0.96	0.97
Stand to sit	-	0.98	0.98	0.97
Sit to stand	-	0.98	0.97	0.98

**Table 3:** The summary of Knee angle measurement comparison (exoskeleton vs. Vicon).

Activity	Speed (m/s)	Correlation Coefficient		
		Subject 1	Subject 2	Subject 3
Level ground Walking	Self-selected	0.95	0.96	0.96
Treadmill walking	0.5	0.96	0.97	0.97
	0.75	0.97	0.96	0.96
	1	0.97	0.96	0.97
	1.25	0.96	0.97	0.96
	Gradual increase (0.5–1.25)	0.96	0.96	0.97
Sit to stand	-	0.98	0.98	0.98
Sit to stand	-	0.98	0.97	0.98

## Data Availability

The data presented in this study are available on request from the corresponding author.
